# Sustained effects of online genetics education: a randomized controlled trial on oncogenetics

**DOI:** 10.1038/ejhg.2013.163

**Published:** 2013-08-14

**Authors:** Elisa JF Houwink, Sarah R van Teeffelen, Arno MM Muijtjens, Lidewij Henneman, Florijn Jacobi, Scheltus J van Luijk, Geert Jan Dinant, Cees van der Vleuten, Martina C Cornel

**Affiliations:** 1 Department of Clinical Genetics, Section Community Genetics, EMGO Institute for Health and Care Research, VU University Medical Center, Amsterdam, The Netherlands; 2 Department of General Practice, School for Public Health and Primary Care, Maastricht University, Maastricht, The Netherlands; 3 Department of Educational Development and Research, Faculty of Health, Medicine and Life Sciences, Maastricht University, Maastricht, The Netherlands; 4 The Dutch College of General Practitioners (NHG), Utrecht, The Netherlands; 5 Department of Education and Resident Training, Maastricht University Medical Centre, Maastricht, The Netherlands

**Keywords:** genetics, health education, primary health care, online CPD, hereditary cancer

## Abstract

Medical professionals are increasingly expected to deliver genetic services in daily patient care. However, genetics education is considered to be suboptimal and in urgent need of revision and innovation. We designed a Genetics e-learning Continuing Professional Development (CPD) module aimed at improving general practitioners' (GPs') knowledge about oncogenetics, and we conducted a randomized controlled trial to evaluate the outcomes at the first two levels of the Kirkpatrick framework (satisfaction, learning and behavior). Between September 2011 and March 2012, a parallel-group, pre- and post-retention (6-month follow-up) controlled group intervention trial was conducted, with repeated measurements using validated questionnaires. Eighty Dutch GP volunteers were randomly assigned to the intervention or the control group. Satisfaction with the module was high, with the three item's scores in the range 4.1–4.3 (5-point scale) and a global score of 7.9 (10-point scale). Knowledge gains post test and at retention test were 0.055 (*P*<0.05) and 0.079 (*P*<0.01), respectively, with moderate effect sizes (0.27 and 0.31, respectively). The participants appreciated applicability in daily practice of knowledge aspects (item scores 3.3–3.8, five-point scale), but scores on self-reported identification of disease, referral to a specialist and knowledge about the possibilities/limitations of genetic testing were near neutral (2.7–2.8, five-point scale). The Genetics e-learning CPD module proved to be a feasible, satisfactory and clinically applicable method to improve oncogenetics knowledge. The educational effects can inform further development of online genetics modules aimed at improving physicians' genetics knowledge and could potentially be relevant internationally and across a wider range of potential audiences.

## Introduction

Although the dramatic surge in the volume and potential applicability of genetics knowledge in medical care is set to continue, there appears to be a marked underuse of this knowledge, in particular among primary-care physicians.^1, 2^ This is probably largely because of physicians lacking sufficient knowledge about genetics for daily practice^3, 4, 5, 6, 7, 8^ and failing to keep up with recent developments in genetic testing.^9^ It is therefore not surprising that there are inadequacies reported in the delivery of genetic services.^10^ In view of the ongoing rapid developments in genetics research, it is important that genomic literacy among healthcare providers be enhanced to ensure optimal translation to health-care delivery of research on common complex diseases, including familial cancers, such as breast and colon cancer. Previous studies have shown that as far as genetics is concerned non-genetic healthcare workers require not only education but also clear guidelines and definitions of their responsibilities.^11, 12, 13^

Continuing Professional Development (CPD) seems the obvious vehicle for remedying deficiencies in practicing physicians' genetics knowledge and skills. In addition, e-learning appears to offer a cost-effective and time-efficient method of keeping physicians informed of new developments. In CPD, e-learning and other modalities (printed educational materials and face-to-face activities) are widely used^14^ and have been shown to be equally effective.^15, 16^ In 2010, the Accreditation Council for Continuing Medical Education in the United States reported online (enduring materials) activities accounted for 28% of all CPD activities, with 4.6 million US physician participants (activity attendees). Online CPD (eCPD) activities represent by far the most popular form of CPD in the United States (40% of all CPD credits).^17, 18^ Between 2003 and 2010, the number of physicians receiving credit for online CPD increased by 800%, compared with an 89% increase for all CPD programs.^18^ These findings and reported improvements in knowledge and clinical decision making following online case vignette courses^19^ suggest that online educational activities can offer ‘a searchable, credible, available on-demand, high-impact source for physicians.'^20^ So far, however, there is a paucity of research into optimizing eCPD and its relevance to everyday primary care, with two small studies evaluating eCPD being methodologically weak and of uncertain clinical significance.^16, 21^ Nevertheless, considering that eCPD is easy to deliver on a large scale and is relatively inexpensive to develop, it is important to determine the feasibility and effectiveness of accredited Genetics eCPD (G-eCPD) in keeping physicians abreast of new genetics developments affecting the delivery of (preventive) cancer care. We therefore conducted a randomized controlled trial (RCT) to investigate the effectiveness and applicability in daily practice of an oncogenetics eCPD module. We aimed to measure the educational outcomes of the module at the first two levels of Kirkpatrick's four-level framework for evaluating educational outcomes^22, 23^ (satisfaction, knowledge and knowledge retention, behavior, and actual practice performance and results^24^). We investigated participant satisfaction (level 1), participants' gain in knowledge about oncogenetics and participants' self-reported application of newly acquired oncogenetics knowledge in daily practice (level 2). To our knowledge, the online module we developed was the first of its kind to be based on assessment of primary-care physicians' educational needs and priorities in relation to genetics knowledge and on core competencies for genetics education. Primary-care physicians' gains in knowledge about oncogenetics in family medicine are expected to improve referral strategies to clinical genetics services and adherence to clinical guidelines. This would increase the feasibility of identification of familial forms of cancer by primary-care physicians, which in turn would improve risk stratification in clinical practice and ultimately reduce morbidity and mortality.

## Materials and methods

### Experimental design

We designed an RCT to assess the outcomes of a G-eCPD module for primary-care physicians at the first two of Kirkpatrick's levels of educational outcomes. The intervention consisted of an oncogenetics eCPD module written by The Dutch College of General Practitioners (NHG; FJ) and the first author of this manuscript (EJFH). Two clinical geneticists and an educationalist (SvL) supported the development of the module. The trial was conducted between September 2011 and March 2012. To control for external effects, a control group was included and, to detect any changes over time, educational outcomes were measured by a pre- and post-test and a retention (6 months) evaluation trial. The study protocol was approved by the ethical review board of the Netherlands Association for Medical Education and the medical ethical review boards of the Maastricht University Medical Center and the VU University Medical Center in The Netherlands. Participation was voluntary and participants gave written informed consent before the start of the trial.

### Study participants

**Figure 1:**
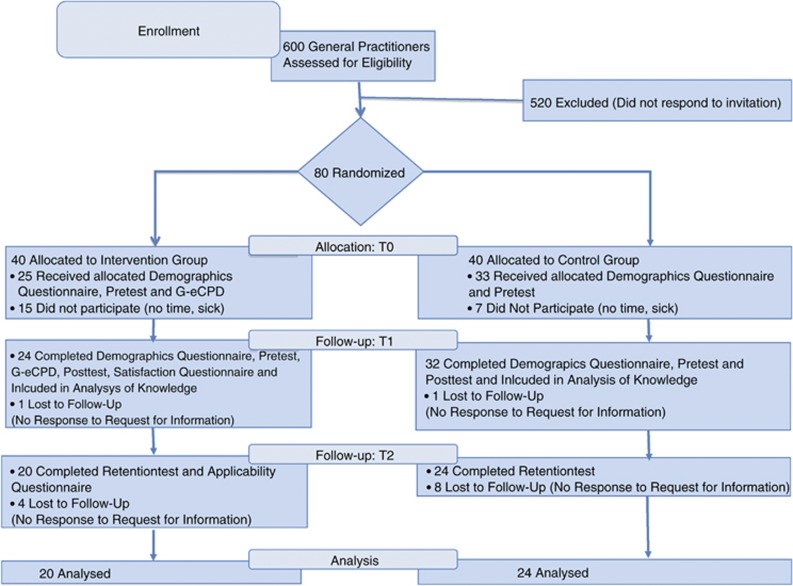
Randomization scheme and participation flow of the online G-eCPD study groups.

General practitioners (GPs) working full time or part time in family practice were eligible for inclusion in the study. Out of 600 Dutch GPs who met the inclusion criterion according to the NHG, 80 responded to participate in the study. Two groups of 40 participants were estimated to be sufficient to detect a medium-to-large effect with a power of 90% and a significance level of 5%.^25^ Figure 1 shows the randomization scheme and participation flow. Participants were recruited by online mailings, informing them about the study and requesting informed consent. CPD accreditation points were awarded for completion of the module, the online knowledge tests and the online questionnaires. A book on genetics in general practice or a book voucher of equal value was offered as an extra incentive.

For sampling and random assignment of participants to the intervention and control group, a pseudo random number generator was used for which the operator was not otherwise involved in the intervention or data analysis. The results of the randomization were communicated to the NHG but not to the researchers.

### Educational design and content

The module contained several didactic components with multimedia presentations, interactive cases with feedback, enabling tools (for referral, family history and online information searches) and other resources, such as step-by-step clinical practice guides and employable NHG guidelines. Common forms of oncogenetic diseases were presented in patient cases. The contents of the module were designed to include 10 items prioritized in a multidisciplinary Delphi study^13^ on core competencies of health professionals^26^ endorsed by the NHG. A multidisciplinary team consisting of FJ and EJFH who wrote the module, educationalists SvL and CvdV, and a clinical geneticist familiar with genetics in primary care, constructed the module and selected practical genetics information on common forms of cancer (such as breast and ovarian cancer, and colon cancer).

The aim of this module was to provide physicians with sufficient knowledge and skills to:

- Identify patients with an inherited predisposition to cancer.
- Draw a family tree as a tool for identifying patients at risk for hereditary cancer.
- Describe the most common types of hereditary cancer (ie, breast cancer and colon cancer) and the likely genetic mutations involved.
- Apply oncogenetics guidelines in identifying patients for whom referral is indicated or not, and find relevant information online.
- Explain the possibilities and limitations of oncogenetic testing.
- Discuss with patients periodic examinations and risk-reducing surgical options that are available to patients with hereditary cancer.

The online module provided access to didactic presentations, such as ‘a clinical genetic cancer consultation in daily practice' interactive cases on breast cancer due to *BRCA* mutations and on colon cancer (eg, Lynch syndrome) due to *APC/*mismatch-repair gene mutations; and enabling tools, such as information about regional possibilities for referral and consultation. The educational sections were presented in the style the NHG usually uses when presenting online CPD modules for optimal recognition. The participants were free to revisit program sections as desired. The module was designed to enable completion within 2 h. The online administration tools afforded monitoring of participant progress, including test and survey completion.

### Data collection

Data were collected using four online instruments: Supplementary Table 1, Questions and Answer Options of the Multiple-choice Knowledge Test; Supplementary Table 2, Satisfaction Questionnaire; Supplementary Table 3, Applicability Questionnaire; and Supplementary Table 4, Demographics and Practice Characteristics Questionnaire. The test questions were based on a validated questionnaire that identified the genetic learning objectives and covered the oncogenetics topics of the G-eCPD.^6^ The instruments were developed and validated in collaboration with content experts (experts in daily clinical genetics, a GP, and an expert in education and questionnaire development) and pilot tested.

The knowledge test contained 20 multiple-choice items. Oncogenetics knowledge was measured as the proportion of correct answers. The satisfaction questionnaire contained 3 items (5-point scale: 1=completely disagree; 5=completely agree) (In the questionnaires, the coding was directed oppositely (1=completely agree, 5=completely disagree) in accordance with the conventions of the NHG. For ease of interpretation in the current paper, the ratings were recoded to comply with international conventions (1=completely disagree, 5=completely agree). related to different aspects of satisfaction, a global grading of the module on a 10-point scale (1=no value; 5=insufficient; 6=sufficient; 8=good; 10=excellent) and a question about the amount of time spent doing the module. The applicability questionnaire contained six five-point Likert scale items about the application of the newly acquired knowledge in daily practice and a multiple-choice question about the application frequency. The demographic survey asked about participants' general characteristics (Supplementary Table 4).

The interventions and measurements were conducted at and between time points T0, T1 and T2 (Table 1). At T0, the intervention and the control group completed the demographics survey and the knowledge test. Between T0 and T1, the intervention group undertook the G-eCPD module, whereas the control group were free to spend the 2-h break any way they wanted, except by doing the module. At T1, both the intervention and the control group again completed the knowledge test in which the question and answer options had been randomly changed to correct for recall bias, and the intervention group completed the satisfaction questionnaire also. At T2, 6 months after T1, retention of knowledge was measured by administering the knowledge test to both groups, whereas the intervention group also completed the applicability questionnaire. After T2, in order to stimulate compliance the online module was made available to the control group, which also completed the satisfaction questionnaire.

### Analysis

Knowledge gain immediately after the module was examined using regression analysis, with Knowledge Test Score at T1 (ScoreT1) as the dependent variable, test score at T0 (ScoreT0) as predictor and Training (0=no, control group; 1=yes, intervention group) as the indicator variable. In order to allow for different slopes for the relation ScoreT1 and ScoreT0, the interaction of Training and ScoreT0 (TrainingxScoreT0) was also included as an independent variable. To improve the interpretation and numerical stability, the independent variable ScoreT0 was centered, and the resulting variable ScoreT0c, equal to ScoreT0−Mean(ScoreT0), was used in the analysis. In a similar analysis, retention of knowledge was analyzed using ScoreT2 as a dependent variable. The regression coefficient corresponding to Training represents the net gain in knowledge (proportion correct) after the intervention, and the standardized regression coefficient indicates the effect size. According to Cohen's categorization, 0.1, 0.3 and 0.5 indicate small, moderate and large effect sizes, respectively.^25^ The final model included only predictors with a statistically significant contribution (*P*<0.05).

The mean test scores and corresponding 95% confidence intervals (95% CIs) for the two groups at T0, T1 and T2 were calculated, and Student's *t*-tests were conducted to determine between-group differences. To determine satisfaction, mean scores, 95% CIs and SD were calculated for the pooled data of the intervention and control groups. For the applicability data (intervention group only), the same statistics were calculated. All statistical analyses were performed using the statistical package SPSS version 19 (SPSS, Chicago, IL, USA).

## Results

### Randomization and dropout comparisons

Of the total of 80 participating physicians (40 intervention group and 40 control group), 44 (20 intervention, 24 control group) completed all the learning activities, knowledge tests and questionnaires (Figure 1). Thirty-six participants were lost to follow-up; 22 did not participate because of time limitation or illness, and 14 did not respond to requests for information.

### Participant characteristics

There were no significant differences between intervention and control group in age, gender, years of experience in primary care, type of practice and practice situation (Supplementary Table 5).

### Knowledge

**Figure 2:**
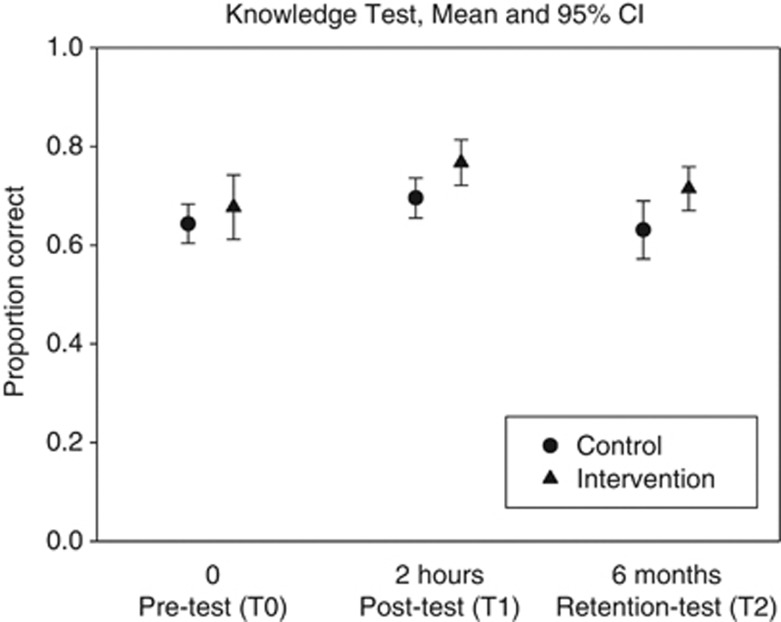
Knowledge test scores (mean and 95% CI) for the control group (circle) and the intervention group (triangle) at T0, T1 and T2, corresponding to pre-, post- and retention measurement, respectively.

Figure 2 presents the results of the pre-test, post-test and retention test. The between-group difference was indifferent or in favor of the intervention group, starting from 0.034 (Student's *t*-test, non-significant, *P*=0.34) at T0, and increasing to 0.072 (*P*<0.05) and 0.084 (*P*<0.05) at T1 and T2, respectively. More precise estimations of knowledge gain were obtained by the regression analysis controlling for between-group differences in ScoreT0 and allowing for an interaction effect of intervention (group) and ScoreT0. The first numerical row of Table 2 shows the regression results for ScoreT1. As the contribution of the interaction was found to be statistically non-significant, the interaction was exluded from the final model, leaving the intercept (Constant), and two additional independent variables (see second row) ScoreT0c, the centered version of the pre-test score (mean ScoreT0=0.66) and the indicator variable Training (0=no, control; 1=yes, intervention). The resulting regression coefficient, the corresponding 95% confidence interval (95% CI; low and high boundary) and the standardized regression coefficient are presented. Value *B*=0.70 for Constant indicates the expected proportion correct in the post-test for a participant in the control group with a ScoreT0 equal to the mean value (0.66). The regression coefficient (0.51) for ScoreT0c indicates the slope of the corresponding regression line for the control group, which was found to be statistically significant as is indicated. The effect of the intervention was found to be statistically significant, amounting to 0.055 on the proportion correct scale; the corresponding value for the standardized regression coefficient was 0.27, indicating an almost moderate effect size. The analysis for ScoreT2 also showed a non-significant interaction and in the final model the intervention effect was found to be 0.079 (standard regression coefficient of 0.31, moderate effect size), implying a further increase of the knowledge effect by 0.024 at 6 months after the intervention.

### Satisfaction and applicability

Table 3 shows the results for satisfaction and applicability. The four satisfaction items had scores of at least 4.1 (95% CI lower boundary not <3.7) and a mean global score of 7.9 (95% CI lower boundary=7.5). The average time spent on the module (124 min) was very close to the recommended time. The applicability scores were more diverse: neutral scores (2.7–2.9) were obtained for self-reported recognition of disease, referral to a specialist and knowledge of possibilities/limitations of genetic testing. The scores on increased knowledge about genetic diseases, concepts and information sources were more positive (3.3–3.8). More than 90% of participants indicated applying newly acquired knowledge at least once a month, and 5% indicated a frequency of at least once a week. No participant reported daily application, and 5% indicated not having encountered any genetic problem in their practice in the last 6 months.

## Discussion

To our knowledge, our study is the first to evaluate the outcomes of an online genetics CPD module at the first two levels of Kirkpatrick's framework, taking oncogenetics as an example. The results indicate that presenting a case-based oncogenetics module in an accessible online learning environment can result in sustained improvement of genetics knowledge for daily medical practice. Other topics, such as cardiogenetics (ie, long QT syndrome or hyperthrophic cardiomyopathy) or diabetes (ie, maturity-onset diabetes of the young), could also be trained in this framework. The findings in the current study indicate that this approach may help to transfer urgently needed genetics knowledge on a broad array of issues, both in primary and secondary care. The participants were satisfied with the module and indicated that they actually applied their newly acquired knowledge in daily practice. However, self-reported applicability aspects focused on practice received neutral scores. This seems to indicate the G-eCPD mainly improved genetics knowledge rather than skills. A live training on oncogenetics may put more emphasis on these performance-oriented aspects reflected in increased consultation skills (ie, recognizing patient with genetic disease, how to refer to a Clinical Genetics center or to be able to explain possibilities/limitations of genetic tests).^27^ Of course, the evaluation of the G-eCPD module should be an ongoing process, which can sustainably help to improve effectiveness. These findings are encouraging for future work in this challenging area of education.

The results indicate that significant knowledge gains of moderate effect size persisted for 6 months after the 2-h educational intervention. This is consistent with reports in the literature that most educational interventions lead to modest-to-moderate improvements in health care.^28^ In addition to knowledge gains, the module showed relatively good cost effectiveness in terms of both finance and time, and it seems likely that it could easily reach large groups of physicians and possibly medical students as well.

### Limitations

A limitation is the fairly large number of non-responders. Selection bias could have been caused by interested GPs who voluntarily participated and received financial incentives. Similar baseline characteristics of the two groups and comparability of the 55% participation rate to those reported for postal surveys among GPs (60% response rate),^29, 30^ however, indicate that the participants were representative of GPs likely to attend oncogenetic training in the future.

It is possible that participating in the oncogenetics training might become a mandatory part of training for all GPs. Participants in the control group had to wait for training content, possibly causing resistance to finish all measurements. No specific reasons, such as fairly long duration of the study for drop out were reported, rather than general attributes (no time and sickness). It is therefore unlikely that self-selection in dropout negatively impacted the validity of the results. Although there were no significant differences in participant characteristics between intervention and control group, the physicians in our study appeared to be more number of women, younger and less experienced, compared with the general profile of Dutch GPs.^31^ This possibly reflects extra interest in genetics and/or using online learning modules by young woman GPs who recently graduated. Whether the results can be generalized within and beyond the Dutch healthcare system needs further investigation.

Although the results show substantive knowledge gains, it might be argued that we did not compare the online module to any other intervention or more traditional methods, such as paper format or live CPD modules. In education evaluation studies published, there may be publication bias: a wide variety of Internet-based interventions show effectiveness in medical education, perhaps leaving negative studies unpublished.^32^ Given recent rapid developments in genetics, there is not enough staff to provide nationwide traditional education activities. A meta-analysis suggested that Internet-based instruction would be similarly effective to non-Internet interventions. Moreover, Internet-based learning is associated with large positive effects compared with no intervention at all.^32^ Our study could therefore be seen as a proof-of-concept evaluation study and further research will be necessary to confirm comparable effectiveness on sustained knowledge.

Although our evaluation of the educational outcomes of the G-eCPD module by the questionnaire on the application of new knowledge in daily practice (Supplementary Table 6) closely approached Kirkpatrick's third level, assessment by observation of actual behavior was absent. We are currently undertaking a study to determine changes in referrals to Clinical Genetics centers after attending the G-eCPD module. Another study we have planned uses standardized patients to evaluate the effectiveness of face-to-face oncogenetics modules in terms of behavioral changes (level 3), such as family history taking and recognizing the need for referrals to the department of Clinical Genetics.^27^ By applying an effective framework for genetics education and measuring outcome of education on various levels of Kirkpatrick, we might be able to initiate a change in organization (approaching level 4) and find barriers to implementation of genetics education.^33^ Despite the limitations of the current study, however, the results suggest that the module presents a promising innovative educational approach in CPD for health professionals. Despite the obvious importance of evaluation at higher Kirkpatrick levels, the results we found for lower outcome levels are also important to build a solid basis for an advanced impact.

### Generalizability

The results of the present study may contribute to the development of genetics educational programs, and online CPD in particular. Online modules once created could potentially reach a large group of physicians in primary and secondary care (non-clinical geneticists, such as oncologists, cardiologists, pediatricians, etc), and across large geographical areas. In addition, possibly those in nursing professions, medical school students and those attending biology classes could benefit from this framework for online genetics education. Costs are likely to be less than multiple face-to-face sessions; however, the time and expertise to create an effective tool is not insignificant.^32^

General practice in the Netherlands is an open-access full-time service, available to all patients with any medical complain or question. As, under the Dutch system, the entire population, irrespective of the presence of disease, is on the list of a GP, optimal continuity of care is guaranteed. If genetic counseling is available in the region, the GP manages most referrals to this service for healthy family members of cancer patients. The GP is therefore likely the first healthcare professional to whom a patient will turn with questions about genetics.

It is also important to consider whether the current results can be generalized to future effects. Obviously, the results are representative for those GPs who participated in the trial on a voluntary basis. It is reasonable to assume that the participants are representative of the group of GPs who, in the future, would be willing to make use of online CPD modules. In other words, the results seem to be generalizable to future users of online CPD modules.

Voluntary participation may have led to self-selection of participants with a special interest in genetics or in clinical leadership qualities. Furthermore, the online module may be made mandatory for all Dutch GPs in the near future. However, it seems plausible that accidental factors, such as time and health problems, rather than specific attributes of participants were responsible for participant dropout. However, specific attributes should be investigated further, for there was a relative high dropout rate (30%).

Studies have reported satisfaction and knowledge gains as a result of online modules on other topics and have suggested that course outcomes may benefit when a course is designed in accordance with a prior educational needs assessment.^15, 19, 34, 35, 36^ The advantages of online CPD have also been broadly discussed and supported.^37^ Various reviews, however, have pointed to heightened effects on physician behavior of multiple interventions compared with single episodic interventions. Multifaceted interventions can tackle several common barriers to change and this combined operation may ultimately lead to improved practice performance. This aspect deserves further study.

### Interpretation

Our online oncogenetics module proved to be a satisfactory and feasible method to achieve urgently needed knowledge improvement in a rapidly evolving field. Web-based genetics education can be a valuable tool to provide physicians, in general, with applicable genomics knowledge,^2^ and the long-term educational effects show great promise with respect to practical and strategy implications.^38^ The results suggest that relatively simple and low-cost online educational activities can have a pivotal role in urgently needed genetic health-care improvement.

## Figures and Tables

**Table 1:** Time table of the RCT

*Instrument*	*Group*	*Time*			
		*0*		*2 h*	*6 months*
		*Pre-test (T0)*		*Post-test (T1)*	*Retention test (T2)*
Knowledge test	Intervention	X^a^	Online oncogenetics training	X	X
	Control	X		X	X
Satisfaction questionnaire	Intervention			X	
Applicability questionnaire	Intervention				X
Demographics questionnaire	Intervention & Control	X			

Abbreviation: RCT, randomized controlled trial.

Time table of the RCT showing scheduled measurement times (columns 3–6), instruments (column 1) and measurements made (indicated with X in columns 3–6) in the intervention and control groups (column 2).

a Measurement made with the instrument indicated in column 1 in the group indicated in column 2.

**Table 2:** Effect of the oncogenetics training (G-eCPD module) on the performance of FPs

*Dependent variable*	*Independent variables*								
		*ScoreT0c*				*Training*			
		*Regression coefficient*			*Standardized regression coefficient*	*Regression coefficient*			*Standardized regression coefficient*
			*95% CI*				*95% CI*		
	*Constant* *Regression coefficient*	*Value*	*Low*	*High*	*Value*	*Value*	*Low*	*High*	*Value*
ScoreT1	0.70***	0.51***	0.30	0.71	0.57	0.055*	0.006	0.103	0.27
ScoreT2	0.64***	0.68***	0.43	0.93	0.62	0.079**	0.022	0.136	0.31

Abbreviations: G-eCPD, Genetic online Continuing Professional Development; CI, confidence interval.

Regression results are shown for immediate gain of performance (ScoreT1) and retention of performance (ScoreT2), using the pre-test score (ScoreT0c) as a covariate and the control group score as a reference.

*P<0.05; **P<0.01; ***P<0.001.

**Table 3:** Satisfaction (intervention+control; *N*=44) and self-reported applicability (intervention only; *N*=20) as a result of training with the G-eCPD module

		*95% CI*		
*Variable*	*Mean*	*Low*	*High*	*SD*
*Satisfaction*				
Would recommend the module to a colleague^a^	4.3	3.9	4.6	1.1
Content of the module is relevant for a GP	4.2	3.9	4.6	1.1
Content of the knowledge test is relevant for a GP	4.1	3.7	4.4	1.0
Global score (1–10)	7.9	7.5	8.3	1.3
Time spent on the module (minutes)	124	115	132	27
				
*Applicability*				
Recognize patient with genetic disease sooner	2.8	2.3	3.3	0.98
Sooner refer to or discuss with a genetic specialist	2.7	2.0	3.3	1.2
More knowledge of possibilities/limitations of genetic tests	2.9	2.4	3.4	0.96
More knowledge of genetic diseases	3.6	3.1	4.1	0.98
More knowledge of basic genetic concepts	3.3	2.8	3.8	0.96
More knowledge of genetic information sources	3.8	3.4	4.3	0.88
				
*Proportion of trainees applying the learned knowledge (%)*				
Daily	0			
Weekly	5			
Monthly	90			
Not (do not meet any genetic problems in our practice)	5			

Abbreviations: CI, confidence interval; G-eCPD, Genetic online Continuing Professional Development; GP, general practitioner.

a If not indicated otherwise, results refer to scores of five-point Likert scale items (1=competely disagree, 5=completely agree).
