# Supplementary material for: Sustained effects of online genetics education: a randomized controlled trial on oncogenetics
Source: Eur J Hum Genet. 2013 Aug 14;22(3):310–6. doi: 10.1038/ejhg.2013.163 (PMC3925286; doi:10.1038/ejhg.2013.163)
Supplement: Supplementary Tables [file ejhg2013163x1.doc]

Online-Only Material contents:

eTable 1. Questions and Answer Options of the Multiple-choice Knowledge Test

eTable 2. Satisfaction Questionnaire

eTable 3. Applicability Questionnaire

eTable 4. Demographics and Practice Characteristics Questionnaire

eTable 5. Demographical and Practice Characteristics of Participants

eTable 6. Evaluation of G-eCPD according to two levels of Kirkpatrick

| **eTable 1.** Questions and Answer Options of the Multiple-choice Knowledge Test[[1]](#footnote-2) |
| --- |
| 1. In an autosomal-dominant inheritance pattern, who can pass on the disorder?    - Men    - Women  - Men and women |
| 1. In which category do the most common disorders with a genetic component fall?    - Monogenetic    - Chromosomal    - Multifactoral |
| **Case Study: Ms. van Aalst**  Ms. van Aalst comes to your practice. Her father’s sister was diagnosed with breast cancer at age 40 and died of it. Her daughter (Ms. van Aalst’s cousin) recently received the same diagnosis at age 35. Answer questions 3, 4 & 5 using Case Study: Ms. van Aalst.   1. What is the degree of the relationship between Ms. van Aalst and the sister of her father: 1^st^ , 2^nd^ , 3^rd^ or 4^th^ ?    - The sister of the father is a first-degree relative of Ms. van Aalst.    - The sister of the father is a second-degree relative of Ms. van Aalst.    - The sister of the father is a third-degree relative of Ms. van Aalst.    - The sister of the father is a fourth-degree relative of Ms. van Aalst. 2. Which possible influence does breast cancer in the family of the father have on the risk of Ms. van Aalst for inherited breast cancer?    - Increased    - Decreased    - None 3. Is there an indication to refer Ms. van Aalst to a clinical genetics department based on the available information?    - No    - Yes |
| **Case Study: Ms. Brederode**  Ms. Brederode comes to your practice. The father of her mother was diagnosed with colorectal cancer and died of it shortly after diagnosis, at the age of 50. The sister of her mother was diagnosed with colorectal cancer at age 39 and died of it a year later. Ms. Brederode is 30 years old and has two children. Answer question 6 using Case Study: Ms. Brederode.   1. Is there an indication to refer Ms. Brederode to a clinical genetics department based on the available information according to CBO-guidelines?    - There is an indication to refer Ms. Brederode to a clinical genetics department    - There is no indication to refer Ms. Brederode to a clinical genetics department    - A decision cannot be made based on the available information. |
| 1. HNPCC (or Lynch syndrome) is form of inherited colon cancer in which one gene plays a great role, i.e. it is a monogenetic subtype. Which portion of colon cancer cases involves a recognizable monogenetic subtype?    - Less than 1%    - Approximately 5%    - Approximately 30%    - Approximately 50% 2. An increased chance of colon cancer caused by one gene mutation exists in other disorders as well. In which of the following disorders is that most likely?    - tuberous sclerosis    - adenomatous polyposis coli    - retinoblastoma    - multiple endocrine neoplasia |
| **Case Study: Ms. Crynen**  Ms. Crynen (28) has received test results from the department of clinical genetics indicating that she has a BRCA2 gene mutation. She considers her family to be complete. Ms. Crynenis now considering her options, such as breast cancer screening, mastectomy, oophorectomy and hormonal treatment. Answer questions 10, 11 &12 based on Case Study: Ms. Crynen.   1. Which of the following breast examinations is the most sensitive in young BRCA2 patients (≤30)?    - Palpation by an experienced surgeon    - Mammagram    - MRI    - Sonogram 2. And if Ms. Crynen had been 40 years old, which breast exam would be the most sensitive:    - Palpation by an experienced surgeon + MRI    - Mammagram + MRI    - Sonogram +MRI    - CT + MRI 3. How should the Family Physician approach the options mentioned by Ms. Crynen?    - The FP should discourage breast cancer screening and encourage postmenopausal oophorectomy.    - The FP should encourage bilateral mastectomy and premenopausal oophorectomy.    - The FP should encourage premenopausal oophorectomy. 4. Ms. Crynen is considering prophylactic oophorectomy. *BRCA2* patients have the following prognosis:    - Following the oophorectomy there will be no menopausal symptoms.    - Following the oophorectomy there will be menopausal symptoms, which can be managed with non-hormonal treatment.    - Hormonal treatment to limit menopausal symptoms should follow the oophorectomy. |
| 1. In comparison to hereditary cancer, sporadic cancer usually develops at an:    - earlier age    - later age |
| 1. Which website presents the general guidelines for referrals to clinical geneticists, for advice related to inherited forms of cancer?    - www.erfelijkheid.nl    - www.oncoline.nl    - www.kankerrichtlijn.nl    - www.erfelijkekanker.nl |
| 1. Multiple patients in the same family are diagnosed with colorectal cancer at a young age (age ≤50). Many of these patients have polyps. In the majority of cases, this indicates a mutation in:    - One of the genes that can cause Lynch syndrome (aka HNPCC)    - The *APC* gene that can cause FAP    - The *BRCA1/ BRCA2* gene |
| 1. Multiple patients in the same family are diagnosed with colorectal cancer at a young age (age ≤50). These patients have few or no polyps. In the majority of cases, this indicates a mutation in:    - One of the genes that can cause Lynch syndrome (aka HNPCC)    - The *APC* gene that can cause FAP    - The *BRCA1/ BRCA2* gene |
| 1. Multiple patients in the same family are diagnosed with endometrial cancer. When a genetic predisposition such as this exists, it indicates a mutation in:    - One of the genes that can cause Lynch syndrome (aka HNPCC)    - The *APC* gene that can cause FAP    - The *BRCA1/ BRCA2* gene |
| 1. Patients can screen themselves for increased risk of inherited breast cancer or colorectal cancer via one of the following websites. This screen provides an immediate initial risk estimation, but cannot determine if hereditary cancer exists. Those who are found to have increased risk are advised to visit their family physician to discuss a referral to a clinical geneticist for further testing. Which website is it?    - www.erfelijkheid.nl    - www.oncoline.nl    - www.kankerrichtlijn.nl    - www.erfelijkekanker.nl |
| 1. In a certain family a genetic disorder is passed by women to their sons and daughters, and by men to their sons and daughters, and the chance of repetition is 25-50%. Which inheritance pattern is most likely?    - autosomal dominant with increased penetration    - autosomal recessive with increased penetration    - autosomal recessive with decreased penetration    - autosomal dominant with decreased penetration |
| 1. In a certain family with an inherited form of cancer, many family members have questions about their chance of getting the cancer. What is the usual form of clinical genetic care in such families?    - The first patient visits the clinical geneticist, the family members are informed via telephone by the clinical geneticist.    - The first patient visits the genetic counselor, the family members are informed via telephone by the clinical geneticist.    - The first patient visits the clinical geneticist, the family members are seen by the genetic counselor.    - The first patient visits the genetic counselor, the family members are seen by the clinical geneticist. |

The order of the questions and possible answers was changed from T0 to T1 to T2.

| **eTable 2.** Satisfaction Questionnaire |
| --- |
| 1. I would recommend this internet PIN to my colleagues. (On a scale of 1=Totally Agree to 5=Totally Disagree, 6= Not applicable/ No opinion.) |
| 2. In general, I judge the topics presented in the internet PIN as relevant for family practice. (On a scale of 1=Totally Agree to 5=Totally Disagree, 6= Not applicable/ No opinion.) |
| 3. In general, I judge the questions presented in the knowledge test as relevant for family practice. (On a scale of 1=Totally Agree to 5=Totally Disagree, 6= Not applicable/ No opinion.) |
| 4. Which grade would you give this internet PIN, on a scale of 1= Bad to 10= Perfect? |
| 5. How much time did you spend on this internet PIN?   - Less than an hour - 1 – 1.5 hours - 1.5 - 2 hours - 2- 2.5 hours - 2.5 - 3 hours - More than 3 hours |
| 6a. Which topic(s) appealed to you the most? (Tick one or more boxes please.)   - Genetic tests - Hereditary breast- and colorectal cancer - Referral and cooperation with specialists - No preference   b. Why? …………………………. |
| 7a. Which topic(s) appealed to you the least? (Tick one or more boxes please.)   - Genetic tests - Hereditary breast- and colorectal cancer - Referral and cooperation with specialists - No preference   b. Why? …………………………. |
| 8. Space for optional extra comments: …………………………. |

| **eTable 3.** Applicability Questionnaire |
| --- |
| 1. I apply the knowledge gained from this internet PIN:   - Daily - Weekly - Monthly - I don’t come across any genetic issues in my practice and therefore do not apply the knowledge from the PIN. |
| 2. I recognize patients with a genetic condition much earlier than I did before I completed the internet PIN. (On a scale of 1=Totally Agree to 5=Totally Disagree, 6= Not applicable/ No opinion.) |
| 3. I refer to or consult with a clinical geneticist much earlier than I did before I completed the internet PIN. (On a scale of 1=Totally Agree to 5=Totally Disagree, 6= Not applicable/ No opinion.) |
| 4. I have more knowledge about the possibilities and limits of genetic testing than I had before I completed the internet PIN. (On a scale of 1=Totally Agree to 5=Totally Disagree, 6= Not applicable/ No opinion.) |
| 5. I have more knowledge about the most common genetics conditions in the Netherlands than I had before I completed the internet PIN. (On a scale of 1=Totally Agree to 5=Totally Disagree, 6= Not applicable/ No opinion.) |
| 6. I have more knowledge about fundamental concepts of genetics than I had before I completed the internet PIN. (On a scale of 1=Totally Agree to 5=Totally Disagree, 6= Not applicable/ No opinion.) |
| 7. I have more knowledge about important sources of information about genetics than I had before I completed the internet PIN. (On a scale of 1=Totally Agree to 5=Totally Disagree, 6= Not applicable/ No opinion.) |
| 8. Space for optional extra comments: …………………………. |

| **eTable 4.** Demographics and Practice Characteristics Questionnaire |
| --- |
| 1. You are:    - Male    - Female |
| 1. Age: …years old |
| 1. Number of years experience as Family Physician: …years |
| 1. In which type of practice do you work?    - Solo practice    - Duo practice    - Group practice    - Community Health Center    - Other |
| 1. Degree of Urbanization of Practice Area:    - Metropolitan area (>100.000 residents)    - City (30.000 - 100.000 residents)    - Small Town (5.000 - 30.000 residents)    - Rural area (<5.000 residents in largest village) |

| **eTable 5.** **Demographical and Practice Characteristics of Participants** | | | |
| --- | --- | --- | --- |
|  | **Control Group (n=24)** | **Treatment Group (n=20)** | **Chi-square/**  **Mann-Whitney Test**  **(P values)** |
| **Sex** |  |  |  |
| Female | 20 | 19 | .23 |
| Male | 4 | 1 |  |
| **Age in years** |  |  |  |
| 30-39 | 13 | 10 | .70 |
| 40-49 | 7 | 5 |  |
| 50-59 | 3 | 5 |  |
| 60-69 | 1 | 0 |  |
| **Professional experience in years** |  |  |  |
| less than 10 | 13 | 12 | .92 |
| between 10 and 19 | 7 | 4 |  |
| between 20 and 29 | 3 | 1 |  |
| between 30 and 39 | 1 | 3 |  |
| **Practice Type** |  |  |  |
| Solo practice | 4 | 0 | .29 |
| Duo practice | 3 | 5 |  |
| Group practice | 4 | 5 |  |
| Community Health Center | 4 | 2 |  |
| Other | 9 | 8 |  |
| **Practice Setting** |  |  |  |
| Metropolitan area (>100,000 residents) | 7 | 4 | .82 |
| City (between 30,000 and 100,000 residents) | 7 | 5 |  |
| Small Town (between 5,000 and 30,000 residents) | 7 | 7 |  |
| Rural area (<5,000 residents in largest village) | 3 | 4 |  |

| **eTable 6.** Evaluation of G-eCPD[[2]](#footnote-3) according to two levels of Kirkpatrick | | |
| --- | --- | --- |
| **Instrument** | **Item description** | **Kirkpatrick level of**  **evaluation** |
| Knowledge test | 20 Multiple Choice items; on knowledge of basic genetic principles, genetic disorders, possibilities and limitations of genetic tests, referral possibilities concerning genetics and most important sources of genetic information | KP[[3]](#footnote-4) 1 and 2;  evaluation of participation and  learners behavior  (effects on knowledge and skills) |
| Satisfaction questionnaire | 3 items on user relevance with a 5-point Likert scale. | KP 1;  evaluation of satisfaction and  opinions of participants |
|  | 1 item global evaluation rating score (1-10). |  |
|  | 1 item on amount of time spent on the online module |  |
|  | 2 items on user opinion on personal preference of the topics with multiple choice options and explanation |  |
| Applicability questionnaire | 1 item on frequency of application of knowledge acquired through the online G-eCPD with a 4-point ordinal scale. | KP 2;  effects on attitudes and  perceptions, knowledge and  skills, effects in change of  behavior and possibly evaluation  on the effects on patient genetic  health |
|  | 6 items on applied knowledge in daily practice (recognizing patients with a common genetic form of cancer, referral to secondary care, knowledge of genetic tests and common genetic diseases, basic genetic terms, usage of important resources of genetic information) with a 5-point Likert scale. |  |
| Demographics and Practice Characteristics Questionnaire | 5 items on age, gender, years of experience as a FP[[4]](#footnote-5), practice situation (urban/rural practice) and practice type (group/single practice). | Not applicable |

1. [↑](#footnote-ref-2)
2. Genetic online Continuing Professional Development [↑](#footnote-ref-3)
3. Kirkpatrick levels of effect of Continuing Professional Development (CPD)

   programs (Kirkpatrick 2006) [↑](#footnote-ref-4)
4. Family Physician [↑](#footnote-ref-5)
